# Long-term outcome after bilateral lung transplantation – a retrospective study from a low-volume center experience

**DOI:** 10.1186/s12893-015-0010-8

**Published:** 2015-03-18

**Authors:** Shun-Mao Yang, Shu-Chien Huang, Shuenn-Wen Kuo, Pei-Ming Huang, Sung-Ching Pan, Jang-Ming Lee, Hong-Shiee Lai, Hsao-Hsun Hsu

**Affiliations:** Department of Surgery, National Taiwan University Hospital and National Taiwan University College of Medicine, No. 7, Chung-Shan South Road, Taipei, Taiwan; Department of Internal Medicine, National Taiwan University Hospital and National Taiwan University College of Medicine, No. 7, Chung-Shan South Road, Taipei, Taiwan

**Keywords:** Lung transplantation, ECMO, Low-volume center

## Abstract

**Background:**

The aim of this study is to review the long-term outcomes of bilateral lung transplantation (BLTx) in our institution and examine the potential issues that may influence outcomes in a low-volume center.

**Methods:**

A retrospective review of BLTx performed in our institution between July 2006 and December 2012 was conducted. Standardized donor selection, procurement, and preservation protocols for brain-dead donors were applied. Measured outcomes were in-hospital mortality and actuarial survival using the Kaplan-Meier method.

**Results:**

Twenty-five consecutive patients (13 male, 12 female) underwent BLTx with a mean age of 41.8 ± 13.5 years. Before LTx, the mean body mass index was 18.3 ± 3.1 kg/m2. Seven of these patients (28%) required oxygen supplementation at rest before LTx, while the remaining patients (72%) required noninvasive mechanical ventilation (n = 6, 24%), invasive mechanical ventilation (n = 9, 36%) or extracorporeal membrane oxygenation (ECMO) (n = 3, 12%). The lung grafts were procured from brain-dead donors with the mean age of 26.8 ± 11.4 year and the best PaO2 / FiO2 ratio of 513 ± 77 before procurement. All cross match results between same-race donors and recipients were negative. The percentage of same-sex matching and CMV mismatching were 64% and 4%, respectively. The mean time listed on the transplant list was 308 ± 261 days. The mean ischemic time for the first and second grafts were 222 ± 62 and 361 ± 67 minutes. During transplantation, 22 (88%) patients depended on ECMO and one (4%) on cardiopulmonary bypass support. All but two patients (82%) were discharged home in good condition; two (8%) patients died within 3 months after BLTx. The cumulative survival rates at 1-, 2-, 3-, and 5-years were 88%, 83%, 72%, and 72%, respectively.

**Conclusions:**

Although the comparatively few annual LTx performed is consistent with the low donation rate, our single-center growing experience demonstrates that good post-lung transplant outcomes can be achieved at a low-volume LTx center.

## Background

Owing to the development of improved immunosuppressants and refinements in surgical technique, lung transplantation (LTx) has become the standard of care for most causes of end-stage lung disease over the last two decades. The first LTx in Taiwan was performed in 1991, but by the end of 2013, the total number of LTx was less than 150 cases due to the shortage of suitable cadaveric lung donors. Currently, there are eight medical centers authorized to perform LTx in Taiwan; however, none of them have been able to reach 10 LTx per year. The official transplantation report published in 2011 by the Taiwan Organ Registry and Sharing Center (TORSC) demonstrated that the national LTx survival rates (or the average survival rates of all LTx centers) from January 2006 to December 2010 in Taiwan were 65% at one year and 56% at 3 years, respectively. These numbers are inferior to the worldwide average data published by International Society for Heart and Lung Transplantation (ISHLT) [1]. This has led some physicians to re-examine the risks and benefits of LTx, leading to delayed referral of potential candidates. In the TORSC report, however, some important issues affecting the LTx outcomes were not well-examined. The national donation rate, the varieties between different transplant centers, the effects of transplant center volume, the urgency-based organ allocation policy, and donor and recipient matching all impact survival after LTx. In an attempt to address the void of information and provide more clarity regarding the current information, we report our single-center experience with long-term outcomes after bilateral LTx (BLTx) and evaluate the effects of several important issue that may influence the long-term outcome of LTx.

## Methods

### Study protocol

We performed a retrospective analysis of consecutive patients who have undergone BLTx at the National Taiwan University hospital from July 2006 to December 2012, excluding heart-lung transplantation. Donor, recipient, and surgical data were extracted from patient charts and the Transplant database. This study was approved by the Institutional Review Board of National Taiwan University Hospital, with the informed consent waived.

### Recipient and donor selection

The criteria for donor and recipient selection were identical to those established by the ISHLT. All donor lungs were retrieved from brain-dead donors. In Taiwan, the preference in donor allocation is given to patients with the highest short-term risk of death. When the waiting-lists were listed on the national organ allocation network, they were categorized into three different status according to the disease severity. The status II meant the patient has been qualified as a waiting list and could wait for a suitable donor. The status IB meant the waiting-list needed to be hospitalized due to disease progress. The status IA meant the waiting-list has already relied on ventilator or ECMO support. The first priority of lung donor allocation was the severity of recipient’s disease, which meant that the more severe the underlying disease, the more preference for organ allocation. Because the total national number of LTx in Taiwan was less than 150 cases, the post-LTx survival did not take into account for organ allocation. The other priorities of organ allocation included the results of cross-matching between donor and recipient, and size matching. Because Taiwan is a small island and the national transplant network run well, we have enough time to perform the cross-matching tests and wait for the results before performing LTx. Therefore, the cross-matching results between donors and recipients were all negative in our LTx patients.

### Lung transplant protocols

During the study period 2006 to 2012, standardized protocols were applied; these have been described previously but are summarized briefly here [2]. All donors were evaluated by our institution’s transplant surgeons. Techniques used to assess lung quality included chest radiography, arterial blood gas, bronchoscopy, and visual inspection. For donor lung procurement, a bolus injection of prostaglandin E1 500μg was administered into the main pulmonary artery immediately before cross-clamping the aorta. The donor lung was perfused with 70 mL/kg of Perfadex (Vitrolife AB, Goteborg, Sweden) anterograde through the main pulmonary artery and 1 liter of Perfadex retrograde through the pulmonary veins. BLTx was carried out through a clamshell incision from the 4^th^ or 5^th^ intercostal space. The intraoperative extracorporeal membrane oxygenation (ECMO) system was routinely set up to provide adequate hemodynamic support during BLTx when the recipient already depended on ventilator or ECMO support before BLTx, or when the recipient could not tolerate pneumonectomy of the first native lung. In general, ECMO was weaned off in the operating room after BLTx. However, if there were signs of severe reperfusion lung edema or acute primary graft dysfunction that did not allow the transplanted lung to function well, ECMO was continued during transport from the operating room to the intensive care unit (ICU). Protective ventilator management with low tidal volumes (6 mL/kg of the donor body weight) and high positive end-expiratory pressure was used postoperatively. Early adequate enteral nutrition was achieved via nasal feeding tube inserted to the proximal jejunum under endoscopic guidance 2 days after LTx. A triple drug regimen, including a calcineurin inhibitor (cyclosporine or tacrolimus), an antimetabolite (azathioprine or mycophenolate mofetil), and corticosteroids, was used for maintenance immunosuppression. The choice of antibiotics was based on the results of sputum culture from donor and recipient. In the early post-transplant period, prophylactic valganciclovir was routinely used to prevent cytomegalovirus. Voriconazole was used for fungus and yeast if infection was suspected.

### Statistical analysis

Continuous data are expressed as the mean with the standard deviation; proportions are represented as numbers (%). Categorical data are presented as a frequency and percentage. Cumulative survival following lung transplantation was determined using the Kaplan-Meier method. Overall survival was determined by the time from BLTx to death or to last follow-up through December 31, 2013.

## Results

### Recipient characteristics

The total cohort included 25 consecutive patients (13 men, 12 women), with a mean age of 41.8 ± 13.5 years (range, 18 ~ 67 years). At the time of LTx, mean recipient body mass index (BMI) was 18.3 ± 3.1 kg/m^2^ (range, 12.9 - 24.1 kg/m^2^); five recipients (20%) were hepatitis B virus (HBV) carriers. Before LTx, 7 (28%) patients required oxygen supplementation at rest, 6 (24%) patients needed noninvasive mechanical ventilator (MV) support, 9 (36%) patients depended on invasive MV support (IMV), and 3 (12%) patients were supported by ECMO combined with IMV. The underlying diseases necessitating transplant included bronchiectasis (n = 7), lymphangioleiomyomatosis (n = 5), bronchiolitis obliterans (n = 4), pulmonary arterial hypertension (n = 3), pneumoconiosis (n = 3), idiopathic pulmonary fibrosis (n = 2), and chronic obstructive pulmonary disease (COPD) (n = 1) (Table 1).

**Table 1:** **Demographic and preoperative characteristics**

**Recipient demographic**	**No. or**	**% or**
	**mean ± SD**	**rang**
Age, year	41.8 ± 13.5	18 ~ 67
BMI, kg/m^2^	18.3 ± 3.1	12.9 - 24.1
Sex Female	12	48%
HBV carrier	5	20%
Respiratory status before LTx		
Under oxygen at rest	7	28%
Noninvasive MV	6	24%
Invasive MV	9	36%
ECMO (+invasive MV)	3	12%
Diagnosis		
Bronchiectasis	7	28%
Not associated with CF	6	24%
Associated with CF	1	4%
LAM	5	20%
BO	4	16%
PAH	3	12%
Pneumoconiosis	3	12%
IPF	2	8%
COPD	1	4%

BMI: Body mass index; HBV: Hepatitis B virus.

MV: Mechanical Ventilator.

ECMO: Extracorporeal membrane oxygenation.

CF: Cystic fibrosis.

LAM: Lymphangioleiomyomatosis; BO: Bronchiolitis obliterans.

PAH: Pulmonary arterial hypertension; IPF: Idiopathic pulmonary fibrosis.

COPD: Chronic obstructive pulmonary disease.

### Donor characteristics

The mean donor age was 27 ± 11 years (range, 15 ~ 53 years). Seven (28%) donors were female and 8 (32%) donors had a smoking history. The causes of brain death were head trauma (n = 18, 72%), cerebrovascular accidents (n = 5, 20%), and other conditions (n = 2, 8%). Before harvest, the best ratio of arterial partial pressure of oxygen (PaO_2_) to 100% fraction of inspired oxygen (FiO_2_) was 513 ± 77 (range, 350 ~ 674) and the mean time dependent on the ventilator was 66 ± 52 hours (range, 23 ~ 252 hours) (Table 2). The transplant variables used to match donors and recipients are presented in Table 2 and included negative cross-match results (100%), same ethnic origin (100%), gender (64%), identical blood group (68%), compatible blood group (32%), and cytomegalovirus (CMV) status (96%).

**Table 2:** **Donor characteristics**

**Donor demographic**	**No. or**	**% or**
	**mean ± SD**	**range**
Donor variables		
Age, years	26.8 ± 11.4	15 ~ 53
Female	7	28%
Cigarette use	8	32%
Cause of brain death		
Head trauma	18	72%
CVA	5	20%
Others	2	8%
Best PaO_2_/FiO_2_ ratio	513 ± 77	350 - 674
Time on ventilator, hours	66 ± 52	23 - 252
Transplant variables		
Cross-match negative	25	100%
Same-race matching	25	100%
Same-sex matching	16	64%
Blood-group matching		
Identical	17	68%
Compatible	8	32%
CMV matching		
D (+), R (+)	24	96%
D (+), R (-)	1	4%

CVA: Cerebrovascular accident.

PaO_2_/FiO_2_: Arterial partial pressure of oxygen/a fraction of inspired oxygen.

CMV: Cytomegalovirus; D (+): Positive CMV result of Donor serum.

R (+): Positive CMV result of recipient serum.

R (-): Negative CMV result of recipient serum.

### Operative and postoperative characteristics

The mean time between placement on the waiting list and transplantation was 308 ± 261 days (range 19 ~ 1064 days). The ischemic times for the first and second grafts were 222 ± 62 minutes (range 125 ~ 337 minutes) and 361 ± 67 minutes (range 253 ~ 463 minutes). ECMO was used during transplantation for 22 (88%) recipients, and 1 recipient (4%) required the heart-lung machine for cardiopulmonary bypass. For maximal utilization of available resources, 13 (52%) donor lungs underwent concomitant resection of unhealthy areas intraoperatively. Seven of 13 donor surgeries were anatomical lobectomies (three for downsizing resection and four for localized pathological present), and the others were volume-reduction surgeries for size mismatching (Table 3).

**Table 3:** **Operative data**

**Demographics**	**No. or**	**% or**
	**mean ± SD**	**range**
Time on waiting list, days	308 ± 261	19 - 1064
Intraoperative		
Ischemic time, minutes		
First graft	222 ± 62	125 - 337
Second graft	361 ± 67	253 - 463
ECMO or CPB support	23	92%
ECMO	22	88%
CPB	1	4%
Concomitant surgical procedures for donor lung	13	52%
lobectomy	7	28%
Other graft size reductions	6	24%

ECMO: Extracorporeal membrane oxygenation.

CPB: Cardiopulmonary bypass.

### Survival

**Figure 1:**
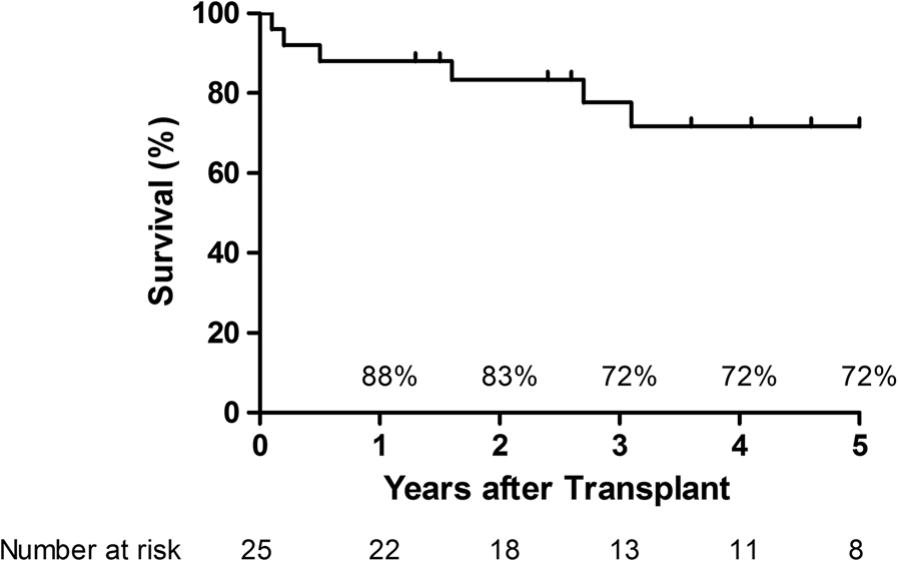
**Kaplan-Meier actuarial survival after bilateral lung transplantation.**

The intensive care stay after BLTx was 45 ± 24 days (range 15 ~ 98 days), and the total hospital stay was 76 ± 45 days (range 21 ~ 206 days). Two recipients died (1 primary graft failure, 1 pulmonary infection) within 3 months; the other recipients (92%) were discharged home without incident and followed up regularly as outpatients. By the end of the study period, four of these discharged recipients had died. The causes of death were pulmonary infection (n = 2), sepsis (n = 1), and bronchiolitis obliterans syndrome (n = 1) (Table 4). The mean survival of all 25 patients was 1272 ± 784 days (range 21 ~ 2544 days). Kaplan-Meier survival for all patients after BLTx at 1-, 2-, 3-, 5- years was 88%, 83%, 72%, and 72%, respectively (Figure 1).

**Table 4:** **Postoperative outcome**

**Demographics**	**No. or**	**% or**
	**mean ± SD**	**range**
Postoperative		
ICU length of stay, days	45 ± 24	15 - 98
Hospital length of stay, days	76 ± 45	21 - 206
Cause of death within less than 90 days	2	8%
Primary graft failure	1	4%
Pulmonary infection	1	4%
Cause of death on day 90 or thereafter	4	16%
Pulmonary infection	2	8%
Sepsis	1	4%
BOS	1	4%
Survival, days	1272 ± 784	21 - 2544

ICU: Intensive care unit.

BOS: Bronchiolitis obliterans syndrome.

## Discussion

This retrospective study presents the long-term outcome of 25 consecutive patients receiving BLTx in a low-volume center. This report demonstrated that the satisfactory outcome can be achieved using the urgency-based organ allocation criteria in a donor-scare country.

According to the TORSC report, the average annual organ donation rate after brain death in Taiwan was around 6.5 per million of population (PMP) in recent years, which is far below that of Spain (35.3 PMP), Belgium (30.1 PMP), Portugal (28.1 PMP), the U.S.A. (25.6 PMP) and France (25 PMP). The low donation rate in Taiwan creates a severe shortage of donor lungs and limits the annual number of LTx. Ample evidence now exists confirming a relationship between surgical outcome and volume of the procedure, which becomes especially important for complex procedures such as transplantation [3-6]. Weiss et al. reported that the cumulative risk of mortality at 30 days, 1 year, and 5 years after LTx was highly dependent on volume. In general, high-volume centers (those centers achieving an average of 20 LTx per year) had superior short- and long-term outcomes, and low-volume centers (those centers achieving less than 10 LTx per year) have alarmingly high mortality rates. In Taiwan, none of the seven authorized LTx centers have been able to reach 10 LTx per year due to the low organ donation rate. Based on these findings, it is easier to understand why the official report of national LTx outcomes in Taiwan is worse than those reported by the ISHLT. Interestingly, some low-volume centers have been able to produce excellent outcomes, implying that factors influencing mortality after LTx are far more complex than simply volume. Weiss et al. speculate that low-volume centers may be able to provide similar outcomes as the high-volume centers if they are equipped with specialized staff, integrated patient support systems, and a culture of excellence. These characteristics provide a benchmark for LTx centers to strive for, no matter the size or volume of the center.

The other important issue that significantly impacts transplant outcomes is the organ allocation system. The new allocation rules implemented in France since 2007, which prioritize allocation of donor lungs to candidates with conditions posing an immediate threat to life, are similar to those in Taiwan. The national LTx report from France demonstrated that the survival results for emergency LTx (ELTx) were worse than those for non-urgent LTx [7]. Survival rates in the ELTx group were 64.5% and 55% at 1 and 2 years, respectively, which were significantly lower than the regular, nonurgent LTx group (77% and 71%, respectively). As with France’s experience, Spain and the United States also have similarly poor outcomes for emergent or urgent LTx [8,9]. According to the United Network for Organ Sharing (UNOS) data, patients on MV or ECMO before LTx had decreased survival after LTx [10]. The 1-year survival associated with ventilator and ECMO support groups were 67.7% and 57.6%, respectively, which were both inferior to those in the high lung allocation score group. Based on these findings, it is reasonable to conclude that this kind of urgency-based allocation policy may contribute to futile and unsuccessful transplantation, especially for low-volume, limited experience LTx centers in donor-scare countries, such as those in Taiwan.

Our report is consistent with the conclusion that lung allocation policy has great influence on the distribution of underlying disease indications for LTx. Although COPD was one of the major diagnoses for LTx in the UNOS and ISHLT reports, only one patient with COPD (4%) received LTx in our study population [1,11]. This unique phenomenon may result from the urgency-based lung allocation system in Taiwan. These allocation rules could potentially make access to LTx more difficult for patients not fulfilling the emergency criteria when donor lungs are scarce, gradually leading to an increase in waiting-list mortality for non-urgent patients, such as those with COPD.

In this study, eighteen (72%) patients were already dependent on MV or ECMO support prior to LTx. In order to provide adequate hemodynamic support during LTx, ECMO was used preferentially as an alternative to cardiopulmonary bypass (CPB). We prefer the ECMO circuit to assist LTx instead of CPB for several reasons. First, modern ECMO technology, including replacement of silicone oxygenators, the introduction of heparin-bonded circuits, and new centrifugal pumps, gives ECMO the capacity to support gas exchange without the need for high-dose heparin administration or anticoagulation therapy. Second, our institution is one of the highest-volume ECMO centers in the world. In our institution since 1994, more than 1,950 patients have been treated with ECMO for sundry causes of cardiopulmonary collapse. Therefore, the refined technical aspects of ECMO combined with the experience using ECMO in our hospital significantly decreases the incidence of major ECMO-attributed complications, including bleeding and peripheral access-related limb ischemia, in our transplant population. Finally, ECMO can provide a bridge to transplantation in patients unresponsive to maximal pulmonary-respiratory support [12-15]. In this report, of 23 patients (92%) receiving BLTx under ECMO support, only one needed to switch to traditional cardiopulmonary bypass (due to severe hemodynamic instability). Furthermore, ECMO allowed 3 (12%) patients cardiopulmonary failure to be stabilized before LTx and thus be successfully bridged (up to 86 days) to BLTx in a high-urgency setting; each of these patients was weaned off ECMO smoothly and discharged without any complications.

The 1-, 3-, and 5-year survival rates after BLTx in this study are similar to those of the Japanese registry report (86%, 79%, and 73%), and are slightly superior to those from UNOS (84%, 66%, 51%) and ISHLT (79%, 64%, 53%) [11,16,17]. Given the small sample size in our study, we can only speculate on the reasons for our improved outcomes. Most likely, a combination of specialized staff, operative technique, and optimal recipient-donor matching (100% cross match and race matching, and only 4% CMV mismatching) is responsible for these outcomes; however, further investigation of these trends is warranted to confirm this speculation.

This study is limited by its single-institution, small sample size, and retrospective nature. Moreover, there was no control group to reduce the influence of selection bias. However, a control group may present ethical challenges because LTx is a promising, feasible and lifesaving procedure for patients with end-stage pulmonary disease.

## Conclusions

Owing to refined surgical technique, adequate perioperative support, and improved postoperative care, we believe LTx can be performed with acceptable post-transplant mortality and satisfactory long-term outcomes in a low-volume center such as ours using the urgency-based organ allocation criteria.

## Abbreviations

BLTx: Bilateral lung transplantation
LTx: Lung transplantation
TORSC: Taiwan Organ Registry and Sharing Center
ISHLT: International Society for Heart and Lung Transplantation
ECMO: Extracorporeal membrane oxygenation
ICU: Intensive care unit
BMI: Body mass index
HBV: Hepatitis B virus
MV: Mechanical ventilator
COPD: Chronic obstructive pulmonary disease
CMV: Cytomegalovirus
PMP: Per million of population
UNOS: United Network for Organ Sharing
CPB: Cardiopulmonary bypass
